# Einstein Model of a Graph to Characterize Protein Folded/Unfolded States

**DOI:** 10.3390/molecules28186659

**Published:** 2023-09-16

**Authors:** Steve Tyler, Christophe Laforge, Adrien Guzzo, Adrien Nicolaï, Gia G. Maisuradze, Patrick Senet

**Affiliations:** 1 Laboratoire Interdisciplinaire Carnot de Bourgogne, UMR CNRS 6303, Université de Bourgogne, 21078 Dijon CEDEX, France; 2 Baker Laboratory of Chemistry and Chemical Biology, Cornell University, Ithaca, NY 14853, USA

**Keywords:** protein folding, intrinsically disordered proteins, graph theory, Kirchhoff index, Wiener index, molecular dynamics

## Abstract

The folded structures of proteins can be accurately predicted by deep learning algorithms from their amino-acid sequences. By contrast, in spite of decades of research studies, the prediction of folding pathways and the unfolded and misfolded states of proteins, which are intimately related to diseases, remains challenging. A two-state (folded/unfolded) description of protein folding dynamics hides the complexity of the unfolded and misfolded microstates. Here, we focus on the development of simplified order parameters to decipher the complexity of disordered protein structures. First, we show that any connected, undirected, and simple graph can be associated with a linear chain of atoms in thermal equilibrium. This analogy provides an interpretation of the usual topological descriptors of a graph, namely the Kirchhoff index and Randić resistance, in terms of effective force constants of a linear chain. We derive an exact relation between the Kirchhoff index and the average shortest path length for a linear graph and define the free energies of a graph using an Einstein model. Second, we represent the three-dimensional protein structures by connected, undirected, and simple graphs. As a proof of concept, we compute the topological descriptors and the graph free energies for an all-atom molecular dynamics trajectory of folding/unfolding events of the proteins Trp-cage and HP-36 and for the ensemble of experimental NMR models of Trp-cage. The present work shows that the local, nonlocal, and global force constants and free energies of a graph are promising tools to quantify unfolded/disordered protein states and folding/unfolding dynamics. In particular, they allow the detection of transient misfolded rigid states.

## 1. Introduction

In spite of significant advances in experimental [1,2,3,4,5,6,7], theoretical [8,9,10,11,12,13,14,15,16,17,18,19], and computational research [20,21,22,23,24,25,26,27,28,29], many questions related to protein folding remain unanswered [19]. In particular, a complete understanding of preferred folding pathways and misfolding and protein aggregation, which are related to neurodegenerative diseases, still remains a challenge. So far, none of these problems can be tackled by current deep-learning and other recent successful computational approaches of protein folding [25], as these methods relate two ensembles of end structures, the linear sequences of amino acids (completely unfolded unstable structures) and the protein folded geometries extracted from an experimental database, and have no reliable information on the ensemble of intermediate structures. It is worth noting that the inclusion of sequence databases in these methods only improves the results by a few percent, emphasizing the importance of information arising from the physical laws: the database of equilibrium experimental structures.

According to Anfinsen’s principle, the equilibrium protein structure (native state in vivo ) is the global minimum of the protein free energy in vitro [8], i.e., it is governed by the second law of thermodynamics. More precisely, the protein equilibrium structure is defined by the amino-acid sequence and the thermodynamical parameters of the environment (T, p, pH) [9]. The protein folding phenomenon is thus cast in thermodynamic terms as a phase transition between an unfolded state and a folded native state. From the Boltzmann law, we know that free energy always involves all microstates. The partition of microstates in folded and unfolded ensembles is at the heart of Landau’s order parameter theory for which the free energy is expanded as a function of one macroscopic parameter that varies between the two phases. This two-state view is challenged by the nanoscopic nature of the macromolecules. Unlike a macroscopic system, there is no sharp unique melting temperature of the transition for a protein, as it depends on the physical property (order parameter) measured and its spatial localization [4,17,18]. Moreover, protein folding may occur through intermediate states or even via a continuum of states (barrierless folding) [6]. A predominant description of protein folding is thus the consideration of an expansion of free energy as a continuous function of order parameter(s): the protein free-energy landscape (FEL) [12]. As for glasses, the protein FEL has multiple local minima [14,30,31,32]. It evolves as a function of temperature, as often pictured as a funnel [12]. The protein FEL concept is essential to understanding the misfolding and aggregation of these heterogeneous polymers. A challenging problem is to define appropriate order parameters to describe the folded, misfolded, unfolded, and intrinsically disordered ensembles of protein structures. The nonfolded state of proteins is not necessarily random, nor does it resemble a Gaussian chain model, and must be characterized. For example, we showed recently that the α-synuclein monomer, a prototypical intrinsically disordered protein involved in Parkinson’s disease, occurs in two distinct disordered states by using an FEL representation based on two order parameters [33]. Therefore, there is still a need to develop useful representations (order parameters) of the folding process based on fundamental laws. The present work aims to develop and test order parameters derived from graph theory [34,35] to contribute to the characterization of protein-disordered ensembles. The theoretical concepts developed in the present work will be tested for protein structures extracted from all-atom molecular dynamics (MD) simulations. Small- and medium-size proteins have been successfully folded using physical laws by all-atom MD simulations [22].

To associate a graph with a protein structure, we represent the amino acids by just a set of points (vertices of the protein graph) together with lines (edges of the graph) joining pairs of these points according to some rules (see Section 3). We select the C${}^{\alpha }$ atom in the protein structure as a vertex representing each amino acid in the graph. For example, the model protein studied here, TRP-cage, will be represented by a graph of 20 vertices. The graph derived from atom positions in a 3D protein structure is hereby called a protein graph (PG). Unlike 3D models, where each C${}^{\alpha }$ atom has a defined position in space and links between the C${}^{\alpha }$ represent pseudo bonds, the relative positions of the vertices and of the shape and length of lines representing edges of a 2D representation of the PG have a priori no significance. The selection of a specific representation of a PG in 2D will depend on some additional descriptors which will serve to cluster the vertices in groups to reveal hidden information in structural or dynamical properties.

The applications of graphs and simplified three-dimensional networks to analyze protein structures and functions have been widely developed [36,37,38,39]. It was established early that graphs representing protein structures share the characteristics of small-world networks [40,41,42,43]. Critical amino acids [44], conserved amino-acids networks [45] in proteins, and signal propagation within the macromolecule were identified by using graphs [42]. Network models were applied to study protein flexibility [46,47], protein unfolding [48], and protein folding pathways [49,50,51]. The complex network of folding pathways can be represented by a graph where each vertex is a microstate or ensemble of microstates, and the edges represent the transitions between them [49].

Here, we show that two topological descriptors of a PG, the Kirchhoff index and the average shortest path between two vertices, can be used to cluster folded and disordered protein structures. Using a linear chain model and statistical physics, we demonstrate that the Kirchhoff index has the physical meaning of the inverse global force constant of the network, and we introduce the local force constant of a vertex, which can be related to Einstein’s seminal model of crystal heat capacity. The free-energy models of the PG are thus defined based on Einstein’s hypothesis and normal mode analysis. As a proof of concept, the present order parameters are used to analyze an all-atom molecular dynamics folding/unfolding trajectory and the ensemble of experimental NMR models of the fast-folder Trp-cage protein. To test the robustness of the findings, the analysis of the topological parameters was repeated for an all-atom molecular dynamics folding/unfolding trajectory of the fast-folder HP-36 protein.

This paper is organized as follows. In Section 2.1, we present the theory based on the analogy between a PG and a 1D chain with harmonic spring force constants. An analytical relation between the Kirchhoff index and the average shortest path length of a graph is derived for a fully unfolded protein. The definitions of the local, nonlocal, and global force constants of a PG and their relation with the Randić resistance of a graph, as well as the definition of the free energies of a PG, are given. In Section 2.2, numerical results are presented for the MD trajectory of Trp-cage. The results for HP-36 are presented in the Section S2 of the Supplementary Materials, as they are similar to those presented in the main text for Trp-cage protein. Technical details on the numerical construction of the PG and on the MD are reported in Section 3. This paper concludes with Section 4.

## 2. Results and Discussion

### 2.1. Theory

#### 2.1.1. Mechanical Interpretation of a Simple, Connected, and Undirected Graph

Here, we introduce the topological equivalence between a simple, connected, and undirected graph and a *linear* chain of atoms with interatomic harmonic potentials.

First, we consider the Hamilonian of the linear chain with *n* atoms in the harmonic approximation:

(1) $$H=\epsilon_{0}+\frac{1}{2}\sum_{i=1}^{n}\sum_{j=1}^{n}\phi_{ij}u_{i}u_{j}$$

where ϕ is the force constant matrix (Hessian), $u_{i}$ and $u_{j}$ are the displacements of the ith and jth atoms of the chain, and $\epsilon_{0}$ is the ground-state energy. Because the chain is linear, the displacements are scalar numbers, which can take positive or negative values. By construction, the chain is connected: there is no atom not linked to another.

Newton’s equations of motion are:

(2) $$\left(\forall i\right):\;f_{i}=m_{i}{\ddot{u}}_{i}$$

where $m_{i}$ is the mass of atom *i*. The force $f_{i}$ is conservative:

(3) $$f_{i}=-\frac{\partial H}{\partial u_{i}}=-\sum_{j=1}^{n}\phi_{ij}u_{j}$$

From Equation (3), one finds:

(4) $$\left(\forall i\right):\phi_{ii}=-\sum_{\begin{array}{c} j=1\\ j\neq i \end{array}}^{n}\phi_{ij}$$

because for a rigid translation, i.e., $u_{i}=U\;\left(\forall i\right)$, all the forces must be zero.

Second, we consider a graph G=(V,E) with vertex set *V* and edge set *E*. The number of vertices in *V* is *n*. We assume the graph to be undirected, simple, and connected. A pair of vertices $v_{i}$ and $v_{j}$ has edge weight $w_{ij}$, which is defined to be 0 if there is no edge between $v_{i}$ and $v_{j}$. Because the graph is assumed undirected, $w_{ij}=w_{ji}$. The adjacency matrix *A* has elements:

(5) $$A_{ij}=w_{ij}\;\mathrm{if}\;\;i\neq j\;\mathrm{and}\;A_{ii}=0$$

The degree $d_{i}$ of the ith vertex is the sum of the weights of all edges having $v_{i}$ as one end:

(6) $$d_{i}=\sum_{j=1,j\neq i}^{n}w_{ij}=\sum_{j=1,j\neq i}^{n}A_{ij}$$

The Laplacian *L* of the graph is defined as usual by:

(7) $$L_{ij}=-A_{ij}\;\mathrm{if}\;\;i\neq j\;\mathrm{and}\;L_{ii}=d_{i}$$

There is a complete equivalence between *L* and ϕ if we interpret the nondiagonal elements of the force constant matrix as proportional to minus the weights of the edges of a graph connecting the atoms, i.e.,

(8) $$\phi_{ij}=-A_{ij}c=-w_{ij}c\;\mathrm{if}\;\;i\neq j$$

where *c* is an arbitrary force constant, which ensures the proper physical dimension of ϕ. Therefore, we have:

(9) ϕ=cL

For a particular case of an unweighted graph for which *A* is a binary matrix (all nonzero weights are equal to 1), the associated linear chain has atoms connected with the same spring force constant *c*. For the particular case of the PG, *A* is binary. The vertices of the PG represent all the C${}^{\alpha }$ atoms of the protein. An edge within the PG represents a contact between two C${}^{\alpha }$ atoms, i.e., they are at a distance in the 3D protein structure shorter than a cut-off radius (see Section 3 for the construction of the PG). The PG Laplacian is thus equivalent to the force constant matrix of a linear chain where the atoms are connected (according to *A*) by the same harmonic spring strength *c*.

The spectral properties of *L* are equivalent (to a constant factor) to the spectral properties of the Hessian (Equation (9)). For a chain of atoms having the same mass, i.e., $\left(\forall i\right):\;m_{i}=m$, the eigenvalues of *L* are related to the vibrational modes of the chain. Indeed, assuming a harmonic solution at the frequency ω for the displacement of the ith atom, i.e., $u_{i}\left(t\right)=u_{i}\left(0\right)e^{i\omega t}$, then ${\ddot{u}}_{i}\left(t\right)=\left(-\omega^{2}\right)u_{i}\left(0\right)e^{i\omega t}$. Using this in Equation (3), we find the following usual eigenvalue equation:

(10) $$\omega^{2}u_{i}\left(0\right)=\sum_{j=1}^{n}\left[\frac{\phi_{ij}}{m}\right]u_{j}\left(0\right)\equiv \sum_{j=1}^{n}B_{ij}u_{j}\left(0\right)$$

The diagonalization of *B* gives the frequencies $\omega_{l}$ and eigenvectors $e_{l}$ of the vibrational modes of the chain:

(11) $$B_{ij}=\sum_{l=1}^{n}\omega_{l}^{2}e_{l}\left(i\right)e_{l}\left(j\right)$$

where $e_{l}\left(i\right)$ is the component of the eigenvector of the ith atom in the lth vibrational mode. We sort the modes by increasing frequency $\omega_{l+1}>\omega_{l}$. The mode 1 corresponds to a translation with $\omega_{1}=0$.

From Equations (9) and (10), the eigenvectors of *L* are identical to those of *B*, and thus the spectral decomposition of *L* is:

(12) $$L_{ij}=\sum_{l=1}^{n}\lambda_{l}e_{l}\left(i\right)e_{l}\left(j\right)$$

with eigenvalues given by:

(13) $$\lambda_{l}=\frac{\omega_{l}^{2}}{\Omega^{2}}$$

with $\Omega^{2}=c/m$.

One has $\lambda_{1}=0$ as expected for a connected graph. The lowest nonzero eigenvalue $\lambda_{2}$ is named the algebraic connectivity, and the eigenvector $e_{2}$ is the Fiedler vector, which can be used to partition the graph. The corresponding vibrational mode 2 of the atomic chain is the mode with the largest wavelength, i.e., the components of its eigenvector are those that fluctuate less along the chain, i.e., less varying among the vertices in a graph.

#### 2.1.2. Thermostatistical Interpretation of Topological Descriptors of a Simple, Connected, and Undirected Graph

We define three descriptors of the thermal fluctuations of a linear chain of atoms with harmonic interatomic potentials and show their equivalence with topological descriptors of a simple, connected, and undirected graph.

The most general solution of the equations of motions is a linear combination of the eigenmodes:

(14) $$u_{i}\left(t\right)=\sum_{l=1}^{n}Q_{l}\left(t\right)\frac{e_{l}\left(i\right)}{\sqrt{m_{i}}}$$

where $Q_{l}\left(t\right)$ are the weights of the modes, the so-called normal coordinates. Using Equations (2) and (3), we have as usual for ∀l≠1:

(15) $$\sqrt{\mu_{l}}{\ddot{Q}}_{l}\left(t\right)+\sqrt{\mu_{l}}\omega_{l}^{2}Q_{l}\left(t\right)=0$$

with the solution $Q_{l}\left(t\right)=Q_{l}\left(0\right)cos\left(\omega_{l}t\right)$ and where $\mu_{l}$ is an arbitrary effective mass. For l=1, $Q_{l}=constant$, and $\omega_{1}=0$. In the microcanonical ensemble (n,V,E) for which the energy *E* is constant, i.e., $Q_{l}\left(0\right)$, it is easy to show that

(16) $$\langle Q_{l}\left(t\right)Q_{l^{\prime }}\left(t\right)\rangle =\delta_{ll^{\prime }}\frac{Q_{l}^{2}\left(0\right)}{2}$$

where $\langle \ldots \rangle =lim_{\tau \to \infty }\frac{1}{\tau }\int_{0}^{\tau }\ldots$ is a time average. In the canonical ensemble (n,V,T), the energy of the microstates are $\frac{P_{l}^{2}}{2\mu_{l}}+\frac{\omega_{l}^{2}Q_{l}^{2}}{2}$, with $P_{l}$ being the momentum associated with mode *l*. Using the equipartition theorem for the chain in thermal equilibrium, we have ∀l≠1:

(17) $${\langle Q_{l}^{2}\rangle }_{T}=\frac{k_{B}T}{\omega_{l}^{2}}$$

where ${\langle \ldots \rangle }_{T}$ is the average over all microstates in the canonical ensemble, *T* is the absolute temperature, and $k_{B}$ is the Boltzmann constant. Because the normal coordinates are statistically independent in the harmonic approximation (as stated by Equation (15), there is no coupling between the modes), thus we have:

(18) $${\langle Q_{l}Q_{l^{\prime }}\rangle }_{T}=\delta_{ll^{\prime }}Q_{l}^{2}$$

and finally, from Equation (14),

(19) $${\langle u_{i}^{2}\rangle }_{T}=k_{B}T\left[\sum_{l=2}^{n}\frac{{\left|e_{l}\left(i\right)\right|}^{2}}{m_{i}\omega_{l}^{2}}\right]=\frac{k_{B}T}{{\hat{k}}_{i}}$$

where we have introduced an effective local force constant (local stiffness) ${\hat{k}}_{i}$. Equation (19) has a clear physical meaning: it represents the statistical fluctuations of the displacement of the *i*th atom in a local harmonic potential with a curvature ${\hat{k}}_{i}$. Summing the atom thermal fluctuations of the entire chain defines an effective global force constant (global stiffness) $\hat{K}$:

(20) $$\sum_{i=1}^{n}{\langle u_{i}^{2}\rangle }_{T}=k_{B}T\left[\sum_{l=2}^{n}\sum_{i=1}^{n}\frac{{\left|e_{l}\left(i\right)\right|}^{2}}{m_{i}\omega_{l}^{2}}\right]=\frac{k_{B}T}{\hat{K}}$$

Equation (20) represents the entire chain as fluctuating in a harmonic ground-state potential of curvature $\hat{K}$. A third effective nonlocal force constant (nonlocal stiffness) can be defined for the thermal fluctuations of a pair of atoms relative to each other:

(21) $${\langle {\left(u_{i}-u_{j}\right)}^{2}\rangle }_{T}=k_{B}T\left[\sum_{l=2}^{n}\frac{{\left|\frac{e_{l}\left(i\right)}{\sqrt{m_{i}}}-\frac{e_{l}\left(j\right)}{\sqrt{m_{j}}}\right|}^{2}}{\omega_{l}^{2}}\right]=\frac{k_{B}T}{{\hat{K}}_{ij}}$$

Equation (21) represents the fluctuations of each pair of atoms ij as if they were in a harmonic potential with curvature ${\hat{K}}_{ij}$. The global force constant can also be defined and measured for actual protein structures, where it is related to the dynamical transition of proteins [52].

The relation between the force constants and the topological descriptors of the corresponding graph is deduced from Equations (19) to (21) by using $m_{i}=m\;\forall i$, the normalization of eigenvectors $\sum_{i=1}^{n}{\left|e_{l}\left(i\right)\right|}^{2}=1$, and Equation (13) for the Laplacian eigenvalues:

(22) $$\begin{array}{r} {\langle u_{i}^{2}\rangle }_{T}=\frac{\gamma }{k_{i}} \end{array}$$

(23) $$\begin{array}{r} \sum_{i=1}^{n}{\langle u_{i}^{2}\rangle }_{T}=\frac{\gamma }{K} \end{array}$$

(24) $$\begin{array}{r} {\langle {\left(u_{i}^{2}-u_{j}^{2}\right)}^{2}\rangle }_{T}=\frac{\gamma }{K_{ij}} \end{array}$$

where $\gamma \equiv \frac{k_{B}T}{c}$ and with the dimensionless local ($k_{i}$), global (*K*), and nonlocal ($K_{ij}$) force constants of the graph defined by

(25) $$\begin{array}{c} \frac{1}{k_{i}}=\sum_{l=2}^{n}\frac{{\left|e_{l}\left(i\right)\right|}^{2}}{\lambda_{l}} \end{array}$$

(26) $$\begin{array}{c} \frac{1}{K}=\sum_{l=2}^{n}\frac{1}{\lambda_{l}} \end{array}$$

(27) $$\begin{array}{c} \frac{1}{K_{ij}}=\sum_{l=2}^{n}\frac{{\left|e_{l}\left(i\right)-e_{l}\left(j\right)\right|}^{2}}{\lambda_{l}} \end{array}$$

The three force constants are related to each other through three sum rules obtained by using the normalization condition $\sum_{i=1}^{n}{\left|e_{l}\left(i\right)\right|}^{2}=1$:

(28) $$\begin{array}{c} \frac{1}{K}=\frac{1}{2n}\sum_{i=1}^{n}\sum_{\overset{j=1}{j\neq i}}^{n}\frac{1}{K_{ij}} \end{array}$$

(29) $$\begin{array}{c} \frac{1}{K}=\sum_{i=1}^{n}\frac{1}{k_{i}} \end{array}$$

(30) $$\begin{array}{c} \frac{1}{k_{i}}=\frac{1}{n}\left[\sum_{j=1}^{n}\frac{1}{K_{ij}}-\frac{1}{K}\right] \end{array}$$

The formulation of the topological descriptors in the context of a chain in thermal equilibrium provides an interesting physical interpretation of the known topological descriptors of a graph as follows. The robustness of a graph is an important concept to measure the quality of a physical network represented by a graph, as in for example a telecommunication system. One measure of the robustness is related to the effective (Randić) resistances of a graph [53]. For a pair of vertices (i,j), this quantity, noted $\Omega_{ij}$, is defined through the Moore–Penrose inverse of the Laplacian, $L^{-1}$:

(31) $$\Omega_{ij}=L_{ii}^{-1}+L_{jj}^{-1}-2L_{ij}^{-1}$$

From the spectral decomposition of the Moore–Penrose inverse of the Laplacian, i.e., $L^{-1}=\sum_{i=1}^{n}e_{l}\left(i\right)e_{l}\left(j\right)/\lambda_{l}$, one immediately find that the nonlocal force constant $K_{ij}$ and the Randić resistance $\Omega_{ij}$ are simply inversely related:

(32) $$\Omega_{ij}=\frac{1}{K_{ij}}$$

Therefore, all the other force constants of a graph can be formulated in terms of $\Omega_{ij}$ because of the sum rules (Equations (28)–(30)). As shown in Randić’s seminal paper, if a connected graph is associated with an electric network with resistances equal to the inverse of the weight $w_{ij}$ between two nodes, $\Omega_{ij}$ represents the effective resistance between the nodes *i* and *j* if a voltage difference is applied between these two nodes. By analogy, if the connected graph is associated with a linear chain with interatomic force constants equal to the weights $w_{ij}$ (normalized by a constant *c*), $K_{ij}$ represents the effective force constant between the atoms *i* and *j* if a couple of forces are applied to this pair of nodes, as explicitly demonstrated in Section S4 of the Supplementary Materials. For a linear chain, the Randić resistance $\Omega_{ij}$ of an atom pair is also exactly its compliance $C_{ij}$ [54] and equal to $1/K_{ij}$. For a three-dimensional elastic network, the compliance is a 3 × 3 tensor representing the elastic response of an atom pair to a couple of forces. A scalar compliance of a pair of nodes of a three-dimensional elastic network, similar to the Randić resistance or nonlocal force constant, can be computed by applying a couple of forces to the atoms in the direction of the vector that joins them [54]. As demonstrated in Section S4 of the Supplementary Materials, this scalar compliance [54] can be related analytically to the tensorial Randić resistance of the atom pair (equal to the inverse of the nonlocal force constant matrix).

Another usual measure of the robustness of a graph is the Kirchhoff index of a graph, Kf, defined by the sum of the eigenvalues of the Moore–Penrose inverse of the Laplacian and is simply the inverse of the global force constant:

(33) $$Kf=\frac{1}{K}$$

The Kf is proportional to the average Randić resistance of the graph. Indeed, from Equation (28), $Kf=1/K=\left(n-1\right)\langle \Omega_{ij}\rangle /4$. For a linear chain, $\langle \Omega_{ij}\rangle$ is also its average compliance.

In the present thermal statistical interpretation of the topological descriptors, each descriptor has the same physical meaning as the stiffness of a specific harmonic potential.

#### 2.1.3. Relation between the Global Force Constant and the Average Shortest Path Length: Analytical Results

The path length between two vertices is defined as the sum of the weights of edges constituting the path. For a binary adjacency matrix, the length of a path between two vertices is the number of edges of the path connecting them. For a path α(i,j) between the vertices *i* and *j*, the length $l_{\alpha (i,j)}$ is

(34) $$l_{\alpha (i,j)}=\sum_{\overset{pairs(r,s)}{\in \;\alpha (i,j)}}w_{rs}=-\frac{1}{c}\sum_{\overset{pairs(r,s)}{\in \;\alpha (i,j)}}\phi_{rs}$$

where (r,s) is an edge of the path α(i,j). The shortest path length between two vertices *i* and *j* is an important topological descriptor. We use the notation $l_{ij}^{0}$:

(35) $$\min{\left\{l_{\alpha (i,j)}\right\}}_{\alpha (i,j)}=l_{ij}^{0}$$

The average over all shortest path lengths between all pairs of vertices of the graph, $\langle l^{0}\rangle$, is another well-studied topological descriptor of the graph robustness and is defined by

(36) $$\langle l^{0}\rangle =\frac{1}{n(n-1)}\sum_{r=1}^{n}\sum_{\overset{s=1}{s\neq r}}^{n}l_{rs}^{0}$$

In Equation (36), the double sum is the so-called graph Wiener index.

As both the Kirchhoff index and the average shortest path length describe the robustness of a network in the literature, a natural question to ask is if and how they are related. An analytical answer can be found for a binary adjacency matrix *A* of a graph for which the first (i=1) and last (i=n) vertices have degree 1, and all the others have degree 2. This graph corresponds to a linear chain with spring force constants between nearest-neighbor atoms only, and the PG is one of a completely unfolded protein (straight polypeptide). For this graph, the average over all shortest path lengths is simply:

(37) $$\langle l^{0}\rangle =\frac{1}{n(n-1)}\sum_{i=1}^{n}\sum_{j=1}^{n}\left|j-i\right|$$

From Equation (37) and simple but tedious algebra (see Section S1 of the Supplementary Materials), one finds

(38) $$\langle l^{0}\rangle =\frac{n+1}{3}$$

The spectral properties of the Laplacian of this graph can be found analytically by analogy with the corresponding linear chain of atoms where $\phi_{ij}=-c$ between nearest-neighbor atoms only. The vibrational frequencies and eigenvectors of such a chain are well known [55] from which we find the eigenvalues of the Laplacian:

(39) $$\lambda_{l}=\frac{\omega_{l}^{2}}{\Omega^{2}}=4\;\sin^{2}\left(\frac{(l-1)\pi }{2n}\right)$$

with l=1,2,3,…n. Using Equations (26) and (39), the global force constant for this graph is given by

(40) $$\frac{1}{K}=\frac{1}{4}\sum_{l=1}^{n-1}\frac{1}{\sin^{2}\left(\frac{l\pi }{2n}\right)}=\frac{n^{2}-1}{6}$$

where the last equality is found as follows. Using $2sin^{2}\left(\frac{l\pi }{2n}\right)=1-cos\left(\frac{l\pi }{n}\right)$, we have

(41) $$\frac{1}{K}=\frac{1}{2}\sum_{l=1}^{n-1}\frac{1}{1-\cos\left(\frac{l\pi }{n}\right)}$$

We observe that $x_{l}\equiv \cos\left(\frac{l\pi }{n}\right)$ are the roots of the derivative of the Chebyshev polynomial of the first kind $T_{n}\left(x\right)=\cos\left(n\theta \right)$ with x=cos(θ). The derivative of $T_{n}\left(x\right)$ is a polynomial of degree n−1: $P_{n-1}\left(x\right)\equiv \frac{dT_{n}\left(x\right)}{dx}\equiv T_{n}^{\prime }\left(x\right)$ [56,57]. Using the chain rule for the derivatives, $P_{n-1}\left(x\right)=\frac{n\sin(n\theta )}{\sin(\theta )}=nU_{n-1}\left(x\right)$, where $U_{n}\left(x\right)$ is the Chebyshev polynomial of the second kind [57]. Then, the sums in the right-hand-side of Equation (41) can be found by using the general rule

(42) $$\frac{d\ln(P_{n-1}\left(x\right))}{dx}=\sum_{l=1}^{n-1}\frac{1}{x-x_{l}}$$

where $x_{l}$ are the roots of $P_{n-1}$. From Equation (42), one has

(43) $$\frac{1}{2}\sum_{l=1}^{n-1}\frac{1}{1-\cos\left(\frac{l\pi }{n}\right)}=\frac{1}{2}\left[\frac{P^{\prime }\left(1\right)}{P(1)}\right]$$

Using l’Hôspital’s rule, one easily finds $P\left(1\right)=n^{2}$, $P^{\prime }\left(1\right)=\frac{n^{4}-n^{2}}{3}$. Inserting these expressions into Equation (43) leads to the announced result in Equation (40). Combining Equations (38) and (40), we derive the analytical relation between *K* and $\langle l^{0}\rangle$ for this particular graph:

(44) $$\frac{1}{K}=\langle l^{0}\rangle \left(\frac{3}{2}\langle l^{0}\rangle -1\right)$$

which shows an inverse relation between the global force constant (1/Kirchhoff index) and the average of the shortest path length.

Equation (44) defines the lowest possible value in the diagram (K,l) of a PG. Indeed, for any PG, there is always a path from vertex *i* to vertex *j* with a length equal to $\left|i-j\right|$ because the corresponding $C^{\alpha }$ atoms form pseudo-bonds at a distance smaller than the cut-off radius defining a contact (see Section 3). Any contact between the $C^{\alpha }$ of amino acids not adjacent in the protein sequence in the 3D protein structure will add an edge that either does not change the length $\left|i-j\right|$ or reduces the length $\left|i-j\right|$. Therefore, the average shortest length given by Equation (37) is the largest possible *among all PGs having the same number of vertices (n amino acids)*. Consequently, the smallest value of *K* is given by Equation (40) and is proportional to $n^{-2}$ for large *n*.

It is worth noting that the graph having the smallest average shortest path length is a complete graph where all vertices are related to all the others by a single edge for which $\langle l^{0}\rangle =1$. This graph is unrealistic for a macromolecule. The eigenvalues of the Laplacian of a complete graph are well known: $\lambda_{1}=0$ and $\lambda_{i}=n\;\forall i\neq 1$. Then, we have for such an extreme case:

(45) $$\frac{1}{K}=1-\frac{1}{n}$$

For a large *n*, the *K* of a complete graph converges to its minimum mathematical value of 1.

#### 2.1.4. Einstein’s Model of a Graph

We build an Einstein model [55] of a simple, connected, and undirected graph by using the mechanical analogy described in Section 2.1.2. The Einstein model is applied here to protein structures recorded every picosecond. On this short timescale, each structure can be considered as fluctuating harmonically on a frozen energy landscape both in the folded and unfolded states. Experimentally, the fluctuations of the vibrational modes of a protein as a function of time can be measured by single-molecule Raman spectroscopy [58]. Following the Einstein model hypothesis, one assumes that each atom i(fori=1,…n) of the linear chain with identical masses and force constants (*c*) has a position fluctuating in a local harmonic potential with a local force constant ${\hat{k}}_{i}$ (Equation (19)) with a local frequency ${\hat{\omega }}_{i}^{2}\equiv \left({\hat{k}}_{i}/m\right)$. Unlike the original Einstein model, one assumes a frequency difference for each atom. The energy of each atom is given by

(46) $${\hat{E}}_{i}={\hat{E}}_{oi}+\left(q+\frac{1}{2}\right)\hbar {\hat{\omega }}_{i}$$

where *q* is the number of vibrational quanta, and ${\hat{E}}_{oi}$ is the potential energy minimum of atom *i*. Assuming thermal equilibrium at a temperature *T* in the (NVT) ensemble, the atom internal energy is [55]

(47) $${\hat{U}}_{i}={\hat{E}}_{oi}+\frac{k_{B}T}{2}{\hat{z}}_{i}\coth\left(\frac{{\hat{z}}_{i}}{2}\right)$$

with ${\hat{z}}_{i}\equiv \frac{\hbar {\hat{\omega }}_{i}}{k_{B}T}$. The atom entropy is

(48) $${\hat{S}}_{i}=k_{B}\left[{\hat{z}}_{i}\left(\frac{1}{\exp({\hat{z}}_{i})-1}\right)-\ln\left(1-\exp(-{\hat{z}}_{i})\right)\right]$$

The classical limit of enthalpy and entropy are found at high temperatures by expanding the exponential around ${\hat{z}}_{i}\ll 1$:

(49) $$\lim_{{\hat{z}}_{i}\ll 1}{\hat{U}}_{i}={\hat{E}}_{oi}+k_{B}T$$

(50) $$\lim_{{\hat{z}}_{i}\ll 1}{\hat{S}}_{i}=k_{B}\left[1-\ln\left({\hat{z}}_{i}\right)\right]$$

The classical limit of the free energy is simply

(51) $$\lim_{{\hat{z}}_{i}\ll 1}{\hat{F}}_{i}={\hat{E}}_{oi}+k_{B}T\ln\left({\hat{z}}_{i}\right)$$

The constant ${\hat{k}}_{i}$ (local frequency ${\hat{\omega }}_{i}$) is associated with a conformation of the chain, i.e., a PG built from the three-dimensional structure of the protein. Assuming some reference conformation of the chain with a free energy ${\hat{F}}_{i}\left(0\right)$ corresponding to a local force constant ${\hat{k}}_{i}\left(0\right)$ and frequency ${\hat{\omega }}_{i}\left(0\right)$, the free-energy difference $\Delta {\hat{F}}_{i}={\hat{F}}_{i}-{\hat{F}}_{i}\left(0\right)$ in the classical limit is

(52) $$\lim_{{\hat{z}}_{i}\ll 1}\Delta {\hat{F}}_{i}=\Delta {\hat{E}}_{i}+k_{B}T\ln\left(\frac{{\hat{z}}_{i}}{{\hat{z}}_{i}\left(0\right)}\right)$$

where the first term is the difference between potential energy minima

(53) $$\Delta {\hat{E}}_{i}={\hat{E}}_{oi}-{\hat{E}}_{oi}\left(0\right)$$

Equation (52) can be simplified

(54) $$\lim_{{\hat{z}}_{i}\ll 1}\Delta {\hat{F}}_{i}=\Delta {\hat{E}}_{i}-\frac{k_{B}T}{2}\ln\left(\frac{{\hat{k}}_{i}\left(0\right)}{{\hat{k}}_{i}}\right)=\Delta {\hat{E}}_{i}-T\Delta {\hat{S}}_{i}$$

where $\Delta {\hat{S}}_{i}$ is the entropy variation, which is the only term depending on the local force constants.

We further make the hypothesis that each atom oscillates independently (as in the Einstein model). Therefore, for *n* amino acids, we have,

(55) $$\lim_{{\hat{z}}_{i}\ll 1}\Delta \hat{F}=\sum_{i=1}^{n}\Delta {\hat{E}}_{i}-\frac{k_{B}T}{2}\sum_{i=1}^{n}\ln\left(\frac{{\hat{k}}_{i}\left(0\right)}{{\hat{k}}_{i}}\right)=\sum_{i=1}^{n}\Delta {\hat{E}}_{i}-\frac{k_{B}T}{2}\ln\left[\prod_{i=1}^{n}\frac{{\hat{k}}_{i}\left(0\right)}{{\hat{k}}_{i}}\right]$$

Thus, from Equation (55), we define the free energy $\Delta F_{local}$ of a PG by

(56) $$\Delta F_{local}=\frac{1}{2}\left\{\epsilon \sum_{i=1}^{n}\left(d_{i}-d_{i}\left(0\right)\right)-\ln\left[\prod_{i=1}^{n}\frac{k_{i}\left(0\right)}{k_{i}}\right]\right\}$$

where the dimensionless parameter ϵ<0 is unknown and controls the enthalpic (potential energy) contribution to the graph free energy. The reference conformation can be the graph examined in the previous section for which *K* is given by Equation (40) (completely unfolded chain) or any other reference, for example, the PG built from the native experimental structure of the protein as in the numerical applications of Section 2.2.

Another formula for the free energy of a graph, $\Delta F_{nonlocal}$, can be built similarly:

(57) $$\Delta F_{nonlocal}=\frac{1}{2}\left\{\epsilon \sum_{i=1}^{n}\left(d_{i}-d_{i}\left(0\right)\right)-\frac{1}{2}\ln\left[\prod_{i=1}^{n}\prod_{j=1}^{n}\frac{K_{ij}\left(0\right)}{K_{ij}}\right]\right\}$$

and finally a coarse-grained expression, $\Delta F_{global}$, is defined:

(58) $$\Delta F_{global}=\frac{1}{2}\left\{\epsilon \sum_{i=1}^{n}\left(d_{i}-d_{i}\left(0\right)\right)-\ln\left[\frac{K(0)}{K}\right]\right\}$$

An important property of the local and nonlocal dimensionless graph free energies of a PG is that the entropic contribution is dominated by the smallest force constants of the graph. For a PG, ΔF is of course $\Delta \hat{F}/k_{B}T$, but Equations (56)–(58) can be applied to any graph where the temperature has no meaning, such as, for example, a communication network.

An alternative to the Einstein model is the graph free energy built from the collective modes of the chain (Equation (15)). Each mode is associated with a collective frequency $\omega_{l}$ (Equation (11)). According to Equation (51), the collective graph free energy is thus defined as:

(59) $$\Delta F_{collective}=\frac{1}{2}\left\{\epsilon \sum_{i=1}^{n}\left(d_{i}-d_{i}\left(0\right)\right)-\ln\left[\prod_{l=2}^{n}\frac{\lambda_{l}\left(0\right)}{\lambda_{l}}\right]\right\}$$

where $\lambda_{l}\left(0\right)$ are the eigenvalues of the Laplacian of the reference conformation (Equation (13)). For the PG of a completely unfolded chain, they are given by Equation (39). If we neglect the degree term, given an ensemble of graphs with the same number of vertices, the one that has the lowest free energy is the one with the smallest *product* of the eigenvalues of its Laplacian.

### 2.2. Topological Analysis of Folding/Unfolding MD Trajectory of Trp-Cage

#### 2.2.1. Two-State Definition

We evaluate and investigate the application of graph force constants and free energies presented in Section 2.1 to folding/unfolding. As a proof of concept, we present here numerical applications for one MD trajectory of the mini-protein: Trp-cage [59]. The Trp-cage is a well-known toy model to study protein folding. This 20-amino-acid peptide is a C-terminal fragment of exendin-4. This construct folds within 4 microseconds in water at physiological pH and exhibits a tightly folded tertiary structure in solution. It consists of a short helix, a 3/10 helix, and a C-terminal poly-proline that packs against a Trp in the alpha helix [59]. The MD trajectory is 500 ns in duration and consists of snapshots calculated on every picosecond when the temperature is 380 K. More details of the MD trajectory are given in Section 3. The strategy is to build the PG of each snapshot and compute the parameters *K* and $<l_{0}>$. In this way, we capture the topological information of the protein structures during the folding/unfolding dynamics.

**Figure 1:**
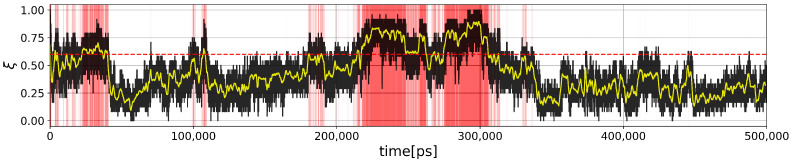
MD trajectory of Trp-cage at 380 K. Time *t* in red (∀t):ξ(t)>0.6. The yellow curve is computed for a moving mean with a window size of 1 ns.

A two-state (folded/unfolded) description of protein folding dynamics hides the complexity of unfolded and misfolded microstates [18]. To decipher the complexity behind these two macrostates, we need first to define them. Many usual global order parameters can be used to partition protein structures in folded and unfolded ensembles. Here, we use the fraction of the native contacts ξ(t) computed for each snapshot at time *t* in the MD trajectories (see Section 3). At time t=0 by construction, ξ(0)=1 and fluctuates below 1 at 380 K (above the unfolding temperature) in the MD trajectory of Trp-cage, as shown in Figure 1. From this figure, we divide the snapshots into a folded state ξ≥0.6 and an unfolded state ξ<0.6. Based on this criterion, we identify an interesting region 100 ns <t< 400 ns where the behavior of descriptors obtained from graph representations can be studied. It contains a folding transition in the first half and an unfolding transition in the next half. It is important to note here that for a protein to function, apart from the *kinetic criterion* of it folding to its native structure, it should also populate its native state for a significant fraction of time which can be mentioned as the *thermodynamic criterion*. Hence, even if we can observe more instances where the fraction of native contacts is above 0.6, the above-mentioned time interval becomes the most important since it pushes the structure to situations where the thermodynamic criterion is favored.

#### 2.2.2. Force Constants and Shortest Path Length

**Figure 2:**
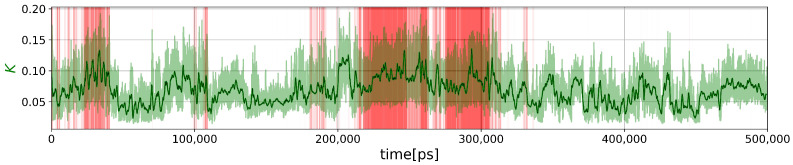
Evolution of the global force constant *K* for the MD trajectory shown in Figure 1. The bold green curve is computed for a moving mean with a window size of 1 ns.

First, we computed the global force constant *K* of the PG as a function of time, as shown in Figure 2.

**Figure 3:**
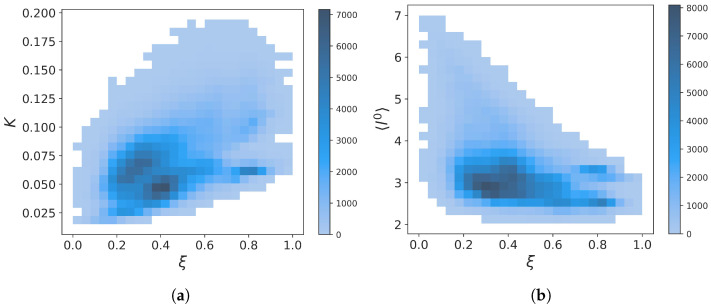
Panels (**a**,**b**) represent respectively the PDF of (ξ,K) values and ($\xi ,\langle l^{0}\rangle$) computed from the trajectory shown in Figure 1.

A visual inspection of the curves shows that the global force constant is somehow related to the fraction of native contacts, but as *K* is more fluctuating than ξ, the Pearson correlation coefficient is not large: 0.4684. As intuitively expected, the time average value of the global force constant of folded structures (ξ≥0.6) $<K_{folded}>=0.0882$ is significantly larger than its value for unfolded structures (ξ<0.6), $<K_{unfolded}>=0.0631$. According to Equation (40), the smallest possible value for Trp-cage is K=0.0150, and the maximum hypothetical value is 1.0526 (Equation (45)). From Figure 2, the minimum and maximum values observed in the MD trajectory are K=0.0150 and K=0.1940, respectively. Although the folded protein is expected to be more rigid than an unfolded polymer chain, disordered or misfolded structures are also expected to be rigid. For example, in the time window 201 ns–208 ns, structures with ξ≈0.4–0.5 have K≈0.12 much larger than $<K_{folded}>$ and twice the value of $<K_{unfolded}>$. Thus, the descriptor *K* contains more information on the unfolded state than the global ξ order parameter. The two-dimensional probability density function (PDF) of the (ξ,K) values computed from the trajectory is represented in Figure 3a and revealed the existence of two unfolded substates at (ξ≈0.3,K≈0.062) and (ξ≈0.4,K≈0.04) and two folded substates at (ξ≈0.8,K≈0.052) and (ξ≈0.8,K≈0.100).

**Figure 4:**
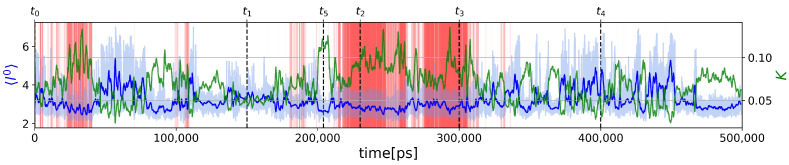
Comparison between the average shortest path length (blue) and global force constant (green) for the MD trajectory shown in Figure 1. The bold green curve is computed for a moving mean with a window size of 1 ns. Times $t_{0}$, $t_{1}$, $t_{2}$, $t_{3}$, $t_{4}$, and $t_{5}$ discussed in the text are indicated.

The time variation of $\langle l^{0}\rangle$ is shown in Figure 4, where it is compared to *K*. The minimum and maximum values observed in the MD trajectory of $\langle l^{0}\rangle$ are 2.0263 and 7.0, respectively. The maximum observed value corresponds to a completely unfolded chain, as predicted by Equation (38). The means of $\langle l^{0}\rangle$ computed for folded (ξ≥0.6) and unfolded structures (ξ<0.6) are 2.8465 and 3.2751, respectively. As expected, the paths in the folded PG are shorter on average. As for *K*, $\langle l^{0}\rangle$ is not significantly correlated with the nativeness characterized by ξ (shown in Figure 1), as the Pearson correlation coefficient is only −0.3920. The variations of $\langle l^{0}\rangle$ thus provide additional information on the different protein substates, as shown by the three local minima of the ($\xi ,\langle l^{0}\rangle$) PDF in Figure 3b.

**Figure 5:**
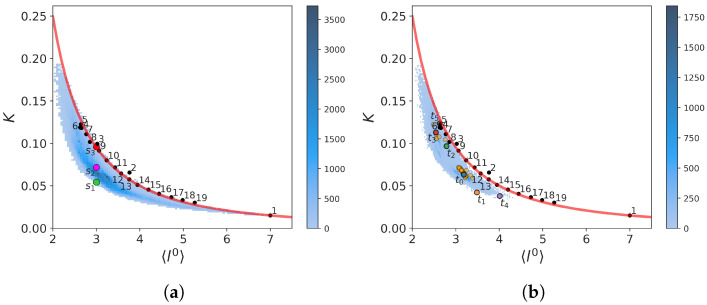

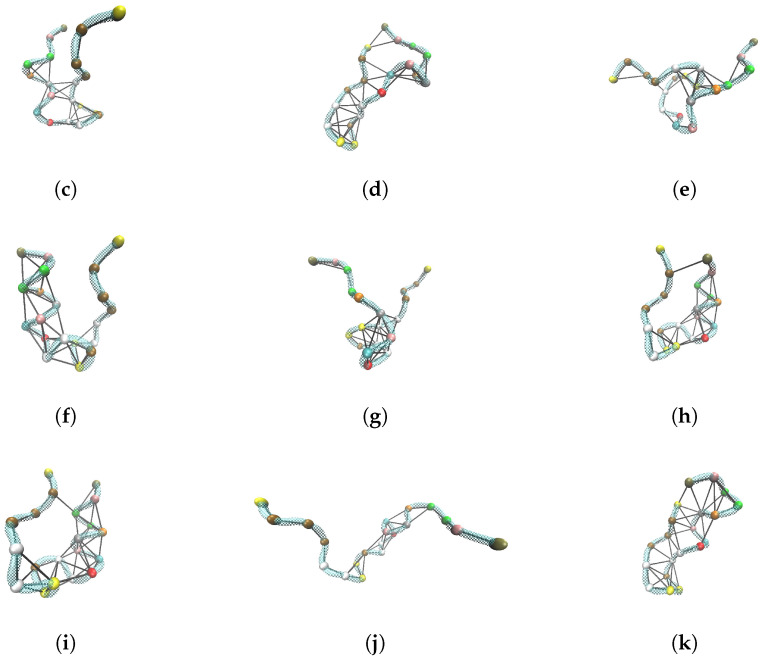
Relationship between *K* and *l* computed for the MD trajectory in Figure 1. Panel (**a**) PDF of (*K*,$\langle l^{0}\rangle$) (blue dots) and pairs of values (*K*,$\langle l^{0}\rangle$) for three selected snapshots named $s_{1}$ (green dot), $s_{2}$ (pink dot), and $s_{3}$ (red dot) with the same value of $\langle l^{0}\rangle$ as discussed in the text. Red line is the result of application of Equation (44). Black dots are the results of model chains with regular long distance spring force constants of different lengths named (20,j=1,2,3…) in the main text. Panel (**b**) PDF of (*K*,$\langle l^{0}\rangle$) from all snapshots with ξ>0.6 (blue). Red line and black dots are as in Panel (**a**). Orange dots are the (*K*,$\langle l^{0}\rangle$) values of the experimental NMR models of Trp-cage (PDB ID: 12lY). Colors dots correspond to the values computed for the snapshots at times $t_{0}$ to $t_{5}$ indicated at Figure 4. Panels (**c**–**k**) are three-dimensional representations of the structures $s_{1}$, $s_{2}$, $s_{3}$ in Panel (**a**) and of the structures at times $t_{0}$, $t_{1}$, $t_{2}$, $t_{3}$, $t_{4}$, $t_{5}$, respectively. The spheres are the positions of the $C^{\alpha }$ atoms, and the tube represents the backbone. The black lines are the contacts considered to build the PG.

Figure 4 clearly shows that $\langle l^{0}\rangle \propto 1/K$ as for a completely unfolded polymer chain (Equation (44)) (Pearson correlation coefficient is −0.8398). However, except for extremal values of $\langle l^{0}\rangle$, a given average shortest path length corresponds to a range of values for *K*, as can be seen from Figure 5a. This can be explained because an intermediate protein size corresponds to a large number of possible conformations with different *K* values. For example, we show three selected structures s1, s2, and s3 (named by increasing *K* value) with the same value $\langle l^{0}\rangle =3$ in Figure 5c–e, respectively. They correspond to graphs with different robustness. In particular, the structures s1 and s2 have N-term and C-term which remain flexible, unlike the s3 structure. The nonuniqueness of the relation between *K* and $\langle l^{0}\rangle$ explains why the PDF of the ($\xi ,\langle l^{0}\rangle$) values computed from the trajectory (Figure 3b) shows only one substate in the unfolded region, whereas the PDF of (ξ,K) (Figure 3a) has two substates.

A striking property of the $\left(K,\langle l^{0}\rangle \right)$ plot is that the ensemble of points draws nearly continuous lower and upper limits. These maximum and minimum values must be degenerated for $\langle l^{0}\rangle =7$ which corresponds to a completely unfolded chain of *n* amino acids according to Equation (38). Indeed, the value of *K* predicted by Equation (44), shown by the black dot with label 1 in Figure 5a, agrees with the MD result. Although Equation (44) was derived for an unfolded chain, we applied it to predict a value of *K* for each value of $\langle l^{0}\rangle$ observed in the MD trajectory. Surprisingly, it predicts nearly perfectly the upper limit of *K* for all the values of $\langle l^{0}\rangle$, as shown in Figure 5a. This unexpected result seems at first glance in contradiction with the fact that for a chain of length *n*, Equation (44) predicts the absolute possible *minimum* value of *K* as explained in Section 2.1.3.

This apparent contradiction is explained as follows. At each value of $\langle l^{0}\rangle$ of the PG of Trp-cage (with n=20 amino acids), we can associate the PG of a completely unfolded shorter protein chain with n<20 amino acids. For example, the value $\langle l^{0}\rangle =3.66$ is the average shortest path length of the PG of an unfolded chain with n=10 vertices according to Equation (38). This unfolded shorter chain can be built from the unfolded chain of n=20 amino acids by eliminating every other amino acid and by connecting the remaining ones by first nearest-neighbor contacts. Therefore, a good approximation of this shorter chain (n=10) by the PG of Trp-cage (n=20) is a structure having contacts only between second nearest-neighbor $C^{\alpha }$ atoms in addition to contacts representing the peptide bonds between the first nearest-neighbor atoms. We name this model (20, 2). The value of *K* of (20, 2) should be close to the minimum value of *K* for a chain with n=10 amino acids predicted by Equation (44). The value of *K* of the (20, 2) chain is shown by the black dot 2 in the $\left(K,\langle l^{0}\rangle \right)$ plot and is indeed very close to the analytical prediction. We have built a series of models of completely unfolded chains (20,j) with contacts only between the third (j=3), fourth (j=4), fifth (j=5), etc., nearest neighbors represented by the black dots numbered, 3, 4, 5, …, respectively. These points follow the predictions of Equation (44) perfectly confirming the reasoning. It can also be seen in Figure 5d that the structure *s*3, close to the upper limit for the value $\langle l^{0}\rangle =3$, corresponds approximately to a three-dimensional structure having third-neighbor contacts only. From a topological point of view, the PG of *s*3 is equivalent to the PG of the (20, 3) structure having a value of *K* close to a chain with n=8. This reasoning explains the predictions of Equation (44) but not why *s*3 is an upper limit for a chain of n=20 for that value of $\langle l^{0}\rangle =3$. This can be understood qualitatively because a PG where each vertex is connected similarly as in (20,j) structures corresponds to a PG where there is no vertex with a low degree, i.e., no weak local force constant which would significantly lower *K* as stated by Equation (30). On the opposite end, as we can see in Figure 5c, the s1 structure with a low *K* has end amino acids connected with only peptide bonds and thus has low local force constants. Although we can figure out the reason for the lower bound in the $\left(K,\langle l^{0}\rangle \right)$, at the time of writing, we have not found an analytical formula to predict it.

It is worth comparing the $\left(K,\langle l^{0}\rangle \right)$ plot extracted for the MD trajectory to the one computed from the 38 experimental NMR models of Trp-cage (PDB code: 1L2Y), as shown in Figure 5b. Surprisingly, the NMR data reveal two distinct groups separated by a gap along the axis $\langle l^{0}\rangle$. The first and second groups are in the regions $2.5<\langle l^{0}\rangle <2.75$ and $3.05<\langle l^{0}\rangle <3.38$, respectively. The first group corresponds to more robust structures with K≈0.10–0.12, whereas the second group has softer structures with K≈0.06–0.07. The native NMR structure used as a reference in the present work (marked $t_{0}$ for which ξ=1 and K=0.0632) is in the second group. Averaging the values of *K* and of $\langle l^{0}\rangle$ of the NMR models gives 0.075 and 3.06, respectively. This is in good agreement with the average values of these quantities computed for folded snapshots (ξ≥0.6 relative to the model chosen at $t_{0}$), which are, respectively, 0.0882 and 3.2751, as mentioned above. The existence of two substates in the native state of Trp-cage was discussed above and are visible in the PDF (K,ξ) (Figure 3a) with a major substate identified as the softest second experimental group and a minor state as the first hardest one. The PDF $\left(\langle l^{0}\rangle ,\xi \right)$ (Figure 3b) also shows the two groups but not with the correct weight as many unfolded structures populated the region of the softer group. Indeed, we recall that the average value $\langle l^{0}\rangle$ computed from unfolded snapshots (ξ<0.6 relative to the model chosen at $t_{0}$) is 2.8465.

The topological descriptors *K* and $\langle l^{0}\rangle$ are global properties of the different protein microstates represented by PGs. A more detailed topological description of these microstates is the sequence of their local force constants $k_{i}$. To illustrate how these sequences vary in the two folding/unfolding transitions (defined here by crossing the limit ξ=0.6 in Figure 1), we selected four representative snapshots in the MD trajectory at $t_{1}=150$ ns (ξ=0.3333,K=0.0422), $t_{2}=230$ ns (ξ=0.8333,K=0.0967), $t_{3}=300$ ns (ξ=0.9167,K=0.1128), and $t_{4}=400$ ns (ξ=0.3333,K=0.0380), as indicated in Figure 4. The snapshots at different times are shown in Figure 5f–k. As we can see in Figure 5b, both structures in the folded state at $t_{2}$ and $t_{3}$ are in the first experimental group (hardest structures). We also selected an unfolded structure at $t_{5}=204$ ns (ξ=0.4583) corresponding to a snapshot with high rigidity, i.e., K=0.1236.

**Figure 6:**
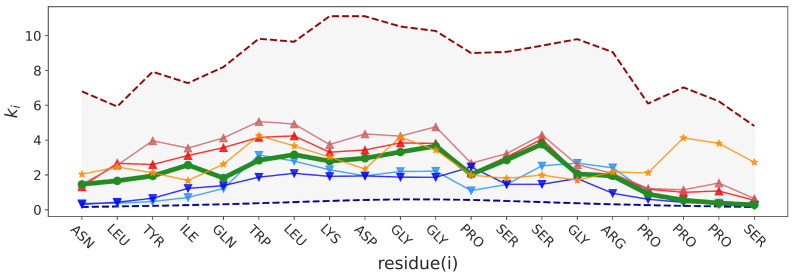
Distribution of the local force constants at times $t_{0}$ (bold green), $t_{1}$ (light blue), $t_{2}$ (red), $t_{3}$ (brown), $t_{4}$ (dark blue) and $t_{5}$ (orange) indicated in Figure 4 and discussed in the text. The gray area limited by dashed lines represents the range of values observed in the MD trajectory.

A representation of sequences of $k_{i}$ is shown in Figure 6. The sequence of $k_{i}$ of the folded structures at times $t_{2}$ and $t_{3}$ are similar. All $k_{i}$ at these times are larger or equal to the values of $k_{i}$ at $t_{0}$ (reference native state). However, this is not sufficient to explain why the native structure has a global force constant nearly twice as small as these two. In fact, the very low $k_{i}$ of PRO18, PRO19, and SER20 at $t_{0}$ decrease *K* significantly (and thus increase the entropy) more for the native structure than for the structures at $t_{2}$ and $t_{3}$. The sums of inverse $k_{i}$ (Equation (30)) from ASN1 to PRO17 at times $t_{0}$, $t_{2}$, $t_{3}$ give a value of *K* equal to 0.1267 [0.0632], 0.1518 [0.0967] and 0.1727 [0.1128] to compare with the values for the complete chain recalled in brackets. The unfolded structures at $t_{1}$ and $t_{4}$ have nearly all their $k_{i}$ smaller than the ones at $t_{0}$, but their low *K* global force constant is mainly due to the very low values of $k_{i}$ at the N-term and C-term regions. Indeed, calculations of the sums of inverse $k_{i}$ of only ASN1, LEU2, TYR3, PRO17, PRO18, PRO19, and SER20 at times $t_{1}$ and $t_{4}$ give values of *K* equal to 0.0620 [0.0422] and 0.0547 [0.0380] which are relatively close to the *K* values of the complete chain recalled in brackets. Low $k_{i}$ at times $t_{0}$ to $t_{4}$ are due to vertices with a low degree, as shown in the representations of the snapshots at different times in Figure 5. On the contrary, the structure at $t_{5}$ has no small $k_{i}$ in the N-term and C-term regions, which explains the strong rigidity of this unfolded state. As can be seen in the representation in Figure 5k, the PG of this snapshot has no vertex with a low degree. In addition, this PG is close to the model structure (20,5) (Figure 3b) and indeed has long-distance contacts. The contributions of residues at the C-term region (PRO17, PRO18, PRO19, and SER20) to *K* explain the large difference of rigidity between the structures at $t_{0}$ and $t_{5}$. Indeed, the calculation of *K* for ASN1 to ARG16 for $t_{0}$ and $t_{5}$ give similar global force constants: 0.1475 [0.0632] and 0.1481 [0.1236], respectively (values for the complete chain are in brackets).

Metastable states competing with the native structure can be related to residual frustration. Frustration in condensed matter physics means that the system cannot simultaneously minimize the competing interactions between its different parts [60]. Proteins evolved in order to minimize frustration, which shapes a funnel free-energy landscape [60]. Although the study of the relations between residual frustration and topological descriptors (global, local, and nonlocal force constants) is far beyond the scope of the present study, we can make some qualitative observations. We computed the local residual frustration configurational index of each amino acid for the native structure of Trp-cage (PDB ID: 1L2Y, model 1) using the protein frustratometer 2 program [61]. The program predicts that amino acids in the N-term (from 1 to 9) are about 20% highly frustrated. This might be qualitatively related to the folded states at $t_{2}$ and $t_{3}$ for which contact between the N-term and C-term stabilizes these non-native folded configurations, as can be seen in Figure 5e,f, respectively. The difference between the sequence of $k_{i}$ of these two configurations with the one of the native structure is also larger in the N-term, as shown in Figure 6.

#### 2.2.3. Calculation of Free Energies Using the Einstein Model

**Figure 7:**
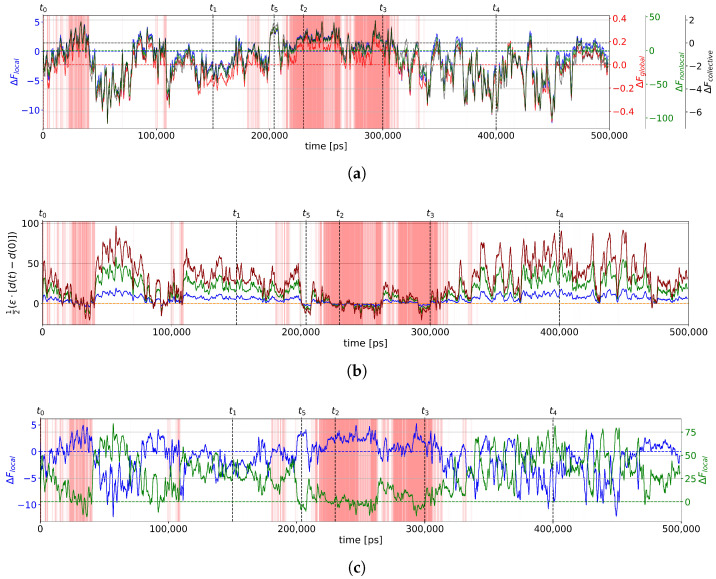
Free-energy graph calculations for the trajectory of Figure 1. (**a**) Local (blue), nonlocal (green), global (red), and collective (black) free energy with ϵ=0. Horizontal dashed lines indicate the zero baselines of the free energies with the corresponding colors. (**b**) Enthalpy term of the free-energy graph with ϵ=−1 (blue), ϵ=−3 (green), and ϵ=−5 (dark red). Horizontal dashed line indicates the zero baseline. $d\left(t\right)\equiv \sum_{i}d_{i}\left(t\right)$ and $d\left(0\right)\equiv \sum_{i}d_{i}\left(0\right)$. (**c**) Local free energy with ϵ=0 (blue) and ϵ=−5 (green). Folded regions are indicated by red vertical lines as in Figure 1. Horizontal dashed lines indicate the zero baselines of the free energies with the corresponding colors.

In the graph free-energy formula derived from the Einstein model, the force constant term is purely entropic (Equation (54)). This contribution is parameter free. The enthalpic part (first term in Equation (56)) depends on an energy scale defined by the single parameter ϵ. As we do not have information on ϵ, we treat it here as a variable. First, we compare the entropic contribution (i.e., for ϵ=0) of the local (Equation (56)), nonlocal (Equation (57)), global (Equation (58)), and collective (Equation (59)) models of the graph free energy in Figure 7a. The local, nonlocal, and collective models agree remarkably with each other with only a change in scale. The coarse-grained global model has the smallest scale and is also very similar to the other models, with a high Pearson correlation coefficient of 0.97 compared to the local model for example. In all models, the entropy change is positive in the folded parts of the MD trajectory, as expected, since the folding reduces possible structural fluctuations. In unfolded parts of the trajectory, the entropy change is mostly negative as expected. There are a few exceptions, for example, times around $t_{5}$. The time parts with positive entropy indicate unfolded very rigid structures. The calculation of the entropic term is thus a means to identify misfolded structures in the trajectory. In Figure 7b, we represent an enthalpic term for different values of ϵ. This term is positive in the unfolded parts of the trajectory, as expected, as the unfolded structures have vertices with a lower degree (fewer contacts), with the structures around $t_{5}$ being an exception. The enthalpic term is small in the folded parts, which indicates that folded structures are on average as connected as the reference structure at $t_{0}$. The enthalpic term is only roughly anticorrelated with the entropic term (the Pearson correlation coefficient between the two terms for the local model is −0.31). We observe structures with a positive entropy (rigid) but with fewer contacts than in the reference folded structure at $t_{0}$ (such as, for example, in the region 80–90 ns). The examination of the enthalpic and entropic parts of the free-energy models permits one to characterize the different rigid misfolded structures. The addition of the two terms is represented for a value of ϵ=−5 in Figure 7c. With this value of ϵ, the structures in time ranges where the folded structure is stable on average (marked red in Figure 1) have zero or negative free energies. The metastable rigid structure at time $t_{5}$ also has negative free energy, whereas most of the unfolded structures have positive free energy.

## 3. Materials and Methods

### 3.1. Contacts and Protein Graph (PG)

Although a PG might be built from the all-atom protein structure, we focus here on a coarse-grained representation of the protein main chain, which only has proven to be useful in describing protein folding [18]. Namely, we represent the protein’s three-dimensional structure by the sole positions of its C${}^{\alpha }$ atoms. Each vertex of the PG thus represents the C${}^{\alpha }$ atom of an amino acid, and the vertices are ordered as in the amino-acid sequences from i=1 to *n*, where *n* is the number of amino acids. An edge between two vertices is drawn if the distance between the two C${}^{\alpha }$ atoms is a contact. A contact is defined as usual for two C${}^{\alpha }$ atoms belonging to nonadjacent amino acids in the protein sequence and which are at a distance in the three-dimensional protein structure below a cut-off radius R=0.6 nm. This typical value includes the peak of the first nearest neighbors of the C${}^{\alpha }$ atoms in folded protein structures. In the present work, a PG is always connected because we add an edge between two C${}^{\alpha }$ atoms, which are nearest neighbors in the amino-acid sequence. These additional edges represent the peptide bonds. The PG is simple, i.e., there is no edge connecting a single vertex (graph loop) or multiple edges between two vertices. We do not make any distinction between the different edges and assume their weight is equal to 1. PG with no contact corresponds to the straight unfolded chain examined in Section 2.1.3 and has the minimum number of edges, i.e., n−1. We define also as usual the native contacts as the contacts present in the experimental folded structure (PDB ID: 1L2Y, model 1). Say $nc_{native}\left(t\right)$, the number of native contacts in the structure of the snapshot at time *t* in the MD trajectory, then we define the fraction of native contacts ξ(t) as follows:

(60) $$\xi \left(t\right)=\frac{nc_{native}\left(t\right)}{nc_{native}^{*}}.$$

where $nc_{native}^{*}$ corresponds to the number of contacts in the experimental native structure. In the MD trajectories studied here, it is also equal to $nc_{native}\left(t=0\right)$ because the initial structure is the experimental one (see Section 3.2). We consider the fraction of native contacts at time *t* to obtain a measure of the structure’s nativeness as a function of time (see text Section 2.2).

### 3.2. Molecular Dynamics Trajectories

The MD trajectory of Trp-cage was generated in a previous unrelated work using an all-atom force field in explicit water at 380 K (above the folding transition temperature) [62]. This MD trajectory was chosen as it clearly shows folding/unfolding events. The MD trajectory is 500 ns in duration and consists of snapshots stored every picosecond (500,000 structures/protein). The initial structure at time t=0 in the MD trajectory is an experimental native structure (PDB ID: 1L2Y, model 1). More details on the MD trajectory can be found in the original paper [62].

### 3.3. Statistics

All statistical calculations (averages, probability densities, Pearson correlation coefficients) were computed from raw data (not from moving average data). The number of bins for both axes in the PDF calculations is 25 for Figure 3 and 100 for Figure 5. The average shortest path length between two vertices was computed with the *average_shortest_path_length* function of the NetworkX Python library [63].

## 4. Conclusions

We emphasize here the main conclusions of the present study and its further extensions. We show that the (K,ξ), $\left(\langle l^{0}\rangle ,\xi \right)$ and $\left(K,\langle l^{0}\rangle \right)$ plots are relevant representations to characterize the diversity of unfolded and folded microstates. The study of $k_{i}$ and *K* as functions of time in an MD trajectory permits the detection of misfolded rigid structures among unfolded conformations. The application of these topological concepts is particularly relevant to characterize the conformations of intrinsically disordered proteins, e.g., α-synuclein [33,64] and will be investigated elsewhere. Topological descriptors and graph free-energy models introduced here permit the characterization of a single simulated or experimental structure at a time. The entropic part is only governed by the force constants computed for the PG associated with a single structure. However, the PG rigidity does not represent of course the full mechanical response of proteins. As a PG is equivalent to a linear chain, it misses the dihedral/rotational degrees of freedom of proteins, which contribute to folding/unfolding transitions [48]. The effect of solvent [65] is also implicit in PG analysis. These degrees of freedom are related to transitions between PGs. Moreover, in protein folding, the stability of structures within a time window must also be considered, i.e., an ensemble of the PGs. An extension of the present theory will include a study of these PG ensembles, their transitions, and the fluctuations of topological descriptors in the folded and unfolded states.

## Data Availability

Data files of the MD trajectory and of the numerical results presented are available on a simple request to the authors.

## Supplementary Materials

The following supporting information can be downloaded at: https://www.mdpi.com/article/10.3390/molecules28186659/s1, Section S1: Mathematical details on the demonstration of Equation (38); Section S2: Topological analysis of folding/unfoldong MD trajectory of HP-36; Section S3: Supplementary figures for HP-36 (PDB ID: 1VII); Section S4: Generalized Randić theorem and relation with compliance

## Abbreviations

The following abbreviations are used in this manuscript:

MD	Molecular Dynamics
PDF	Probability Density Function
