# Supplementary material for: Einstein Model of a Graph to Characterize Protein Folded/Unfolded States
Source: Molecules. 2023 Sep 16;28(18):6659. doi: 10.3390/molecules28186659 (PMC10536427; doi:10.3390/molecules28186659)
Supplement: Supplementary file 1 [file molecules-28-06659-s001.zip › molecules-2586723-supplementary.pdf]

# Supplementary Materials: Einstein Model of a Graph to Characterize Protein Folded/Unfolded States

**Steve Tyler, Christophe Laforge, Adrien Guzzo, Adrien Nicolai, Gia G. Maisuradze and Patrick Senet ***

## S1. Mathematical Details on the Demonstration of Equation (38) of the Main Text

We demonstrate explicitly Equation (38) of the main text. We start from Equation (37):

$$\left\langle l^0 \right\rangle = \frac{S}{n(n-1)} \tag{S1}$$

where

$$S \equiv \sum_{i=1}^{n}\sum_{j=1}^{n}|j-i| = \sum_{i=1}^{n}\sum_{j=1}^{i}(i-j) + \sum_{i=1}^{n}\sum_{j=i+1}^{n}(j-i) \tag{S2}$$

$$S = \sum_{i=1}^{n}\left\{\sum_{j=1}^{i} i - \sum_{j=1}^{i} j + \sum_{j=i+1}^{n} j - \sum_{j=i+1}^{n} i\right\} \tag{S3}$$

To evaluate $S$, we use the two following well-know equations

$$\sum_{m=1}^{n} m = \frac{n(n+1)}{2} \tag{S4}$$

$$\sum_{m=1}^{n} m^2 = \frac{n(n+1)(2n+1)}{6} \tag{S5}$$

From Equation (S4), we have

$$\sum_{m=i+1}^{n} m = \frac{n(n+1)}{2} - \sum_{m=1}^{i} m = \frac{n(n+1)}{2} - \frac{i(i+1)}{2} \tag{S6}$$

Using Equations (S4), (S5) and (S6) in Equation (S3), we find

$$\begin{aligned} S &= \sum_{i=1}^{n}\left\{i^2 - \frac{i(i+1)}{2} + \frac{n(n+1)}{2} - \frac{i(i+1)}{2} - (n-i)i\right\} && \text{(S7)}\\ &= \sum_{i=1}^{n}\left\{\frac{n(n+1)}{2} - i(n+1) + i^2\right\} && \text{(S8)}\\ &= \frac{n^2(n+1)}{2} - \frac{n(n+1)^2}{2} + \frac{n(n+1)(2n+1)}{6} && \text{(S9)}\\ &= \frac{n(n+1)(n-1)}{3} && \text{(S10)} \end{aligned}$$

Finally, we deduce Equation (38)

$$\left\langle l^0 \right\rangle = \frac{n+1}{3} \tag{S11}$$

## S2. Topological Analysis of Folding/Unfolding MD Trajectory of HP-36

The main results found for Trp-cage are robust and does not depend so much on the details of the chosen folding/unfolding trajectory. To show this, we repeated the analysis for a MD trajectory of HP-36 which is another well-known fast folder protein. This is the 36-residue thermostable helical subdomain of the F-actin-binding headpiece domain of

chicken villin. The HP-36 is the smallest domain of a naturally occurring protein that folds cooperatively to a compact helical native state. As for the Trp-cage, the MD trajectory is 500 ns of duration and consists of snapshots calculated on every picosecond at the temperature 380 K. The initial structure at time $t = 0$ in the MD trajectory is the average experimental native structure measured by NMR (PDB ID: 1VII) after relaxation in explicit solvent. More details of the MD trajectory are given in Ref. [1]. We found similar results to those presented in the main text for the analysis of the Trp-cage MD trajectory. Here we summarize the main results.

At time $t = 0$ by construction, $\xi(0) = 1$ and fluctuates below 1 at 380 K (above the unfolding temperature) in the MD trajectory of HP-36 as shown in Figure S1. As for Trp-cage, we divide the snapshots in a folded state $\xi \geq 0.6$ and an unfolded state $\xi < 0.6$. The HP-36 spent less time in the unfolded state than Trp-cage. The unfolded state of HP-36 also is less pronounced than for Trp-cage, i.e. most of $\xi$ values remain above 0.4.

Fluctuations of $K$ on the picosecond and nanosecond time-scales are presented in Figure S2. The fluctuations of $K$ and of $\langle l^0 \rangle$ as a function of time are compared in Figure S3. They are anti-correlated with a Pearson correlation coefficient of -0.8892 similar to the one computed for the Trp-cage trajectory. The minimum and maximum values of $K$ are 0.0083 and 0.0984, respectively. The Equation (40) of the main text predicts a minimum value for $K$ of 0.0046. This means that HP-36 does not unfold completely in the trajectory. As for Trp-cage, the time average values of $K$ in the folded state (0.0362) is larger than in the unfolded state (0.0300). It is worth noting that HP-36 is softer than Trp-cage as its average, minimum and maximum values of $K$, are less than half those of Trp-cage. It is worth noting that both Equations (40) and (45) show than $K$ decreases with $n$.

The values of $\langle l^0 \rangle$ vary between 2.7619 and 8.3127. As HP-36 does not unfold completety, the observed maximum value is lower than the one predicted by Equation (38) of the main text which is 12.3333. As for Trp-cage, the average value of $\langle l^0 \rangle$ in the folded state (4.3577) is smaller than one in the unfolded state (4.4490). The difference between these two values is smaller than for Trp-cage as HP-36 explores less extended states in the MD trajectory.

As for Trp-cage, $K$ and $\langle l^0 \rangle$ are not correlated with $\xi$. The Pearson correlation coefficient between $\xi$ and $K$ is 0.2491 and between $\xi$ and $\langle l^0 \rangle$ -0.0567. These numbers are lower than for Trp-cage. The Probability Density Function (PDF) of $(\xi, K)$ computed from the trajectory is represented in panel (a) of Figure S4 and shows two folded substates ($\xi \approx 0.8 - 0.9$, $\xi \approx 0.7$) and a weak unfolded state ($\xi \approx 0.5 - 0.6$). These states are clearly visible in the PDF of $(\xi,\langle l^0 \rangle$ [Figure S4 (b)]. The variations of $K$ and $\langle l^0 \rangle$ provide additional information on the microstates explored in the folded and unfolded states for a given value of $\xi$. As for Trp-cage, we observe several compact and rigid transient structures with a global force constant much larger than $\left\langle K_{folded} \right\rangle$. For the HP-36 trajectory, these structures are found both in the unfolded and folded states. For example, at time $t_5$ in Figure S3, $K$ is 0.0808 compared to $\left\langle K_{folded} \right\rangle = 0.0362$ and $\langle l^0 \rangle = 3.0143$ compared to $\left\langle l^0_{folded} \right\rangle = 4.3577$. In the unfolded region 200 ns $< t <$ 230 ns, one observes compact rigid misfolded structures with $K \approx 0.06$ twice larger than $\left\langle K_{unfolded} \right\rangle = 0.03$ (Figure S2).

The PDF $(K,\langle l^0 \rangle)$ are shown in Figure S5 (a) and (b). As can be seen, the global force constant varies for a given value of the average shortest path length as for Trp-cage. For example, we show three selected structures s1, s2 and s3 (named by increasing $K$ value) with the same value of $\langle l^0 \rangle = 4$ in Figure S5 (c), (d) and (e), respectively. They correspond to graphs with different robustness. In particular, the structure $s1$ has C-term which remains flexible on the opposite of $s2$ and $s3$ structures. The stiffer structure $s3$ has a contact between the C-term and N-term on the contrary to the two others.

As for Trp-cage, the ensemble of points in the $(K,\langle l^0 \rangle)$ plot draws nearly continuous lower and upper limits. The upper limit is nicely predicted by Equation (44) of the main text as in Figure 5 for Trp-cage. The explanation of this surprising result is identical for

HP-36 and Trp-cage. For each value of $\langle l^0 \rangle$ of HP-36 PG (with $n = 36$ amino acids), there is a completely unfolded shorter protein chain with $n < 36$ amino acids which has a similar value of $\langle l^0 \rangle$. This shorter chain can be approximated by a complete chain ($n = 36$) with contacts only between second, third, etc nearest-neighbors (in addition to the peptide bonds which are always present). As for Trp-cage, we built series of models of completely unfolded chains $(36, j)$ with contacts only between second ($j = 2$), third ($j = 3$), fourth ($j = 4$), fifth ($j = 5$)... nearest-neighbors represented by the black dots in Figure S5 numbered, 2, 3, 4, 5..., respectively. These points follow the predictions of Equation (44) perfectly confirming again the reasoning.

We examined the sequence of the local force constants $k_i$. To illustrate how these sequences vary in the folding/unfolding events, we selected four representative snapshots in the MD trajectory at $t_1 = 100$ ns ($\xi = 0.8167, K = 0.0678$), $t_2 = 220.5$ ns ($\xi = 0.55, K = 0.0344$), $t_3 = 350$ ns ($\xi = 0.5167, K = 0.1373$) and $t_4 = 425$ ns ($\xi = 0.917, K = 0.0329$) indicated at Figure S3. The snapshots at different times are shown in Figure S5 (f) to (k). We selected also a folded structure at $t_5 = 13$ ns ($\xi = 0.85$) corresponding to a snapshot with high rigidity, i.e. $K = 0.081$. The structure at $t_0$ corresponds to ($\xi = 1, K = 0.0315$). The sequences of $k_i$ of the folded structures at times $t_0$ and $t_4$ are nearly identical. The rigid structures at $t_1$ and $t_5$ have similar sequences of $k_i$ but which are very different from the one of the native structure at $t_0$ even if the nativness at $t_1$ and $t_5$ is large and not so different from the one at $t_4$. This clearly indicates that the sequence of local force constants as well as the global force constant of proteins are better descriptors of their nativeness as they allow to detect misfolded states with a large fraction of native contacts. As it can be seen in Figure S5 (d) and (k), the misfolded states correspond to transient structures with contacts between their N-term and C-term. The configurations in the unfolded state at $t_2$ and $t_3$ have different sequences of $k_i$. The global force constant at $t_2$ is closer to $\left\langle K_{folded} \right\rangle$ than $\left\langle K_{unfolded} \right\rangle$ with $k_i$ in the N-term and C-term larger than those of the native structure at $t_0$ . At $t_2$ the protein is in fact misfolded as it can be seen in Figure S5 (e). The structure at $t_3$ is soft as expected for an unfolded protein and indeed the molecule is in an extended conformation as shown in Figure S5 (i).

We compared the entropic contribution (i.e. for $\epsilon = 0$) of the local [Equation (56) of the main text], nonlocal [Equation (57) of the main text], global [Equation (58) of the main text] and collective [Equation (59) of the main text] models of the graph free-energy in Figure S7 (a). As for Trp-cage, the local, nonlocal, global and collective models agree remarkably to each other with only a change of scale. The global model has the smallest scale and is very similar to other models (for example, the Pearson correlation coefficient with the local model is 0.97 as for Trp-cage). As for Trp-cage, in all models the entropy change is positive in the folded parts of the MD trajectory as expected since the folding reduces possible structural fluctuations. For the HP-36, the collective free-energy obeys less strictly to this rule and is negative between 130 and 180 ns although its variation is highly correlated with the nonlocal free-energy. Short excursions of all free-energy models to negative values in the shaded red area, as just before the first unfolded region 200 ns $< t <$ 230 ns, correspond in fact to repeated short times unfolded states represented by thin white lines hardly visible on the plot. In unfolded parts of the trajectory, the entropy change is mostly negative, as expected, as shown in the region 300 ns $< t <$ 380 ns. As for Trp-cage, there are exceptions with positive entropy in the unfolded region pointing to compact misfolded structures with large $K$. This occurs for example in the unfolded region 200 ns $< t <$ 230 ns for the local, nonlocal and global free-energies. The protein is misfolded which explains the positive entropy contribution.

In Figure S7 (b), we represent enthalpic term for different values of $\epsilon$. This term is positive and large in the unfolded parts of the trajectory as expected since the unfolded structures have vertices with a lower degree. The enthalpic term is small in the folded parts which indicates that folded structures are in average as connected as the reference structure at $t_0$ with a few exceptions as at $t_5$ because the structure is misfolded and rigid

with a large number of contacts. The enthalpic term is only roughly anti-correlated with the entropic term (the Pearson correlation coefficient between the two terms for the local model is -0.45). The examination of enthalpic and entropic parts of the free-energy models permits the characterization of the different rigid misfolded structures. The addition of the two terms is represented for a value of $\epsilon = -5$ at Figure S7 (c). With this value of $\epsilon$, the structures in time ranges where the protein is unfolded (white regions in Figure S1) have large positive free-energies. But with this value of $\epsilon$ some folded regions according to the criterion $\xi > 0.6$ have also positive free-energies as between 130 ns and 200 ns, a region preceding the first unfolding region of the MD trajectory. We observe a drift of $\xi$ to lower values in this time region indicating the non-stationarity of the folded state in this time interval.

## S3. Supplementary Figures for HP-36 (PDB ID: 1VII)

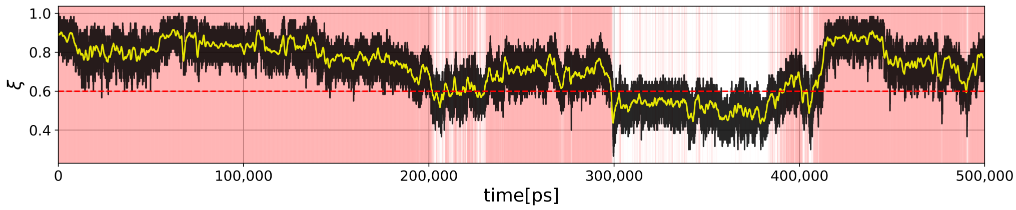


**Figure S1.** MD trajectory of HP-36 at 380K. Time $t$ in red $(\forall t) : \xi(t) > 0.6$. The yellow curve is computed for a moving mean with a window size of 1 ns.

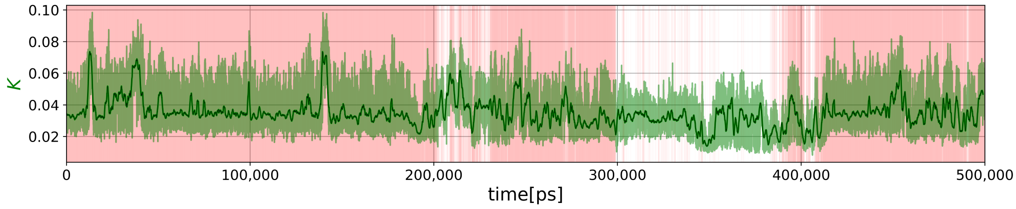


**Figure S2.** Evolution of the global force constant $K$ for the MD trajectory shown in Figure S1. The bold green curve is computed for a moving mean with a window size of 1 ns.

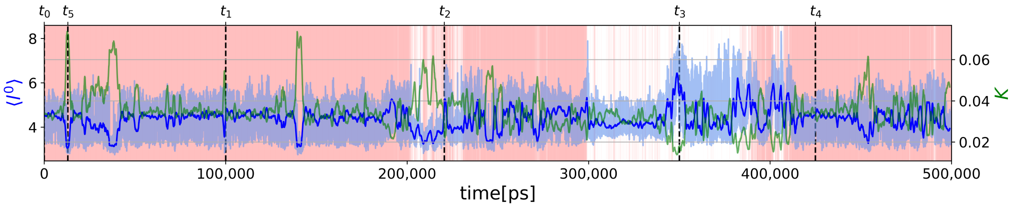


**Figure S3.** Comparison between the average shortest path length (blue) and global force constant (green) for the MD trajectory shown in Figure S1. The bold green curve is computed for a moving mean with a window size of 1 ns. Times $t_0$, $t_1$, $t_2$, $t_3$, $t_4$, $t_5$ discussed in the text are indicated.

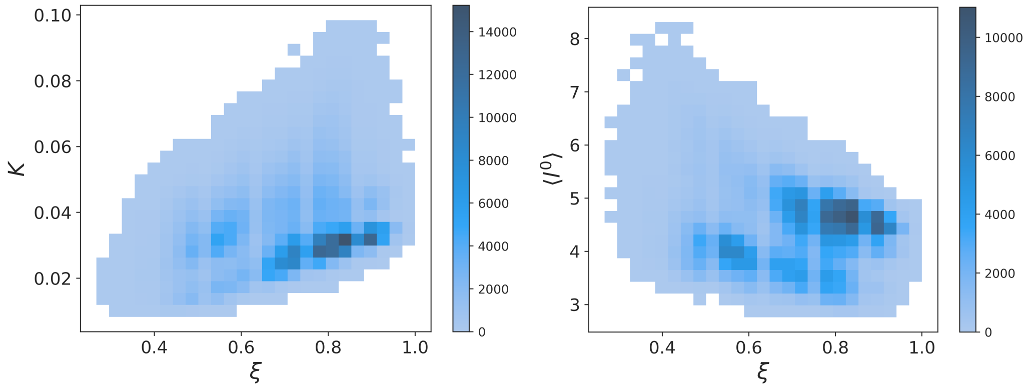


**(a)** **(b)**

**Figure S4.** Panels (a) and (b) represent respectively the PDF of $(\xi, K)$ values and $(\xi, \langle l^0 \rangle)$ computed from the trajectory shown in Figure S1.

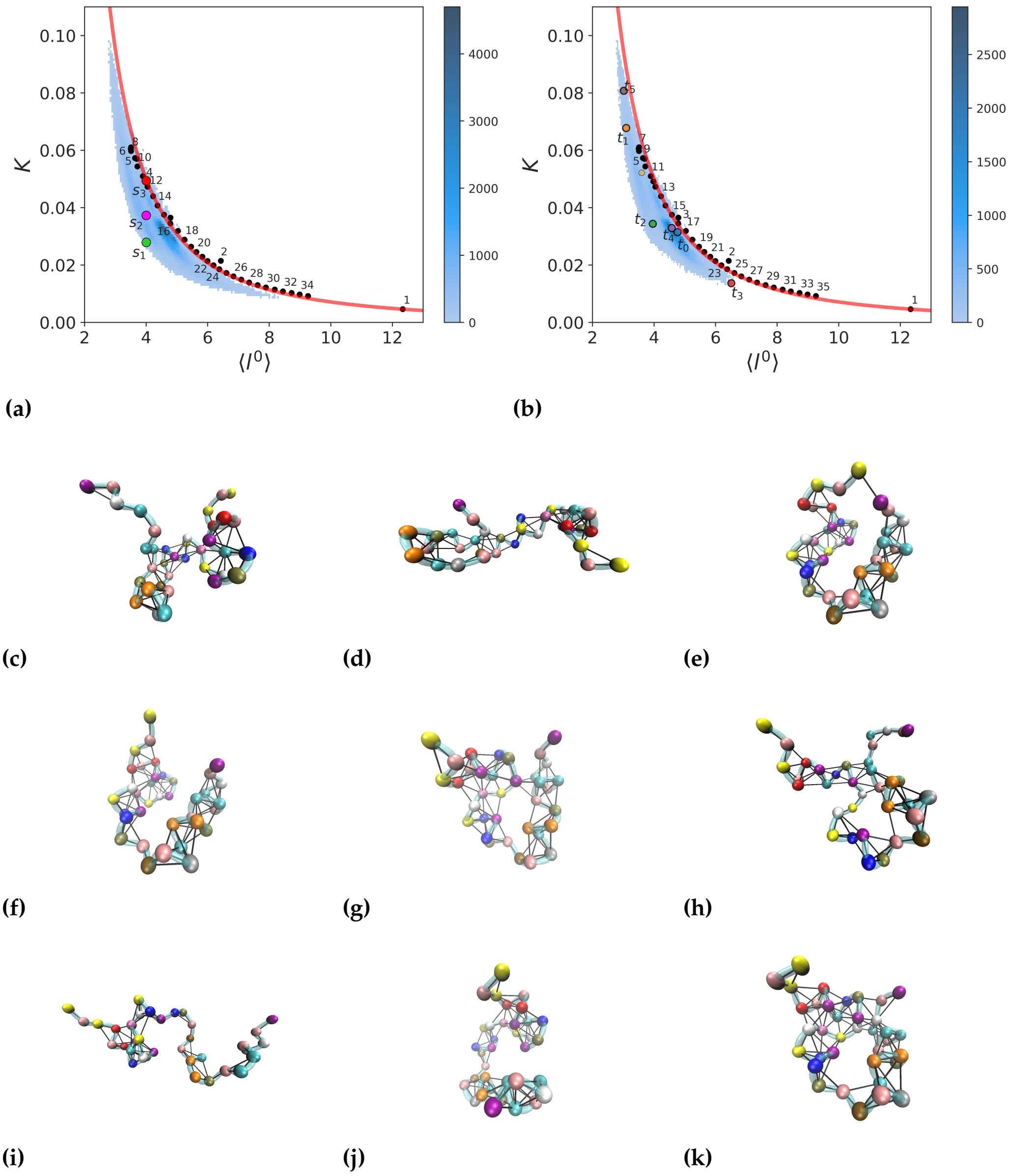


**Figure S5.** Relationship between $K$ and $l$ computed for the MD trajectory in Figure S1. Panel (a) PDF of $(K,\langle l^0\rangle)$ (blue dots) and pairs of values $(K,\langle l^0\rangle)$ for three selected snapshots named $s_1$ (green dot), $s_2$ (pink dot), and $s_3$ (red dot) with the same value of $\langle l^0\rangle$ as discussed in the text. Red line is the result of application of Equation (44) of the main text. Black dots are the results of model chains with regular long distance spring force constants of different lengths named $(36, j = 1, 2, 3...)$ in the text. Panel (b) PDF of $(K,\langle l^0\rangle)$ from all snapshots with $\xi > 0.6$ (blue). Red line and black dots are as in Panel (a). The orange dot is the $(K,\langle l^0\rangle)$ value of the experimental NMR average structure (PDB ID: 1VII). Colors dots correspond to the values computed for the snapshots at times $t_0$ to $t_5$ indicated at Figure S3. Panels (c),(d),(e),(f),(g),(h),(i),(j) and (k) are three-dimensional representations of the structures $s_1$, $s_2$, $s_3$ in Panel (a) and of the structures at times $t_0$, $t_1$, $t_2$, $t_3$, $t_4$, $t_5$, respectively. The spheres are the positions of the $C^\alpha$ atoms and the tube represents the backbone. The black lines are the contacts considered to build the PG.

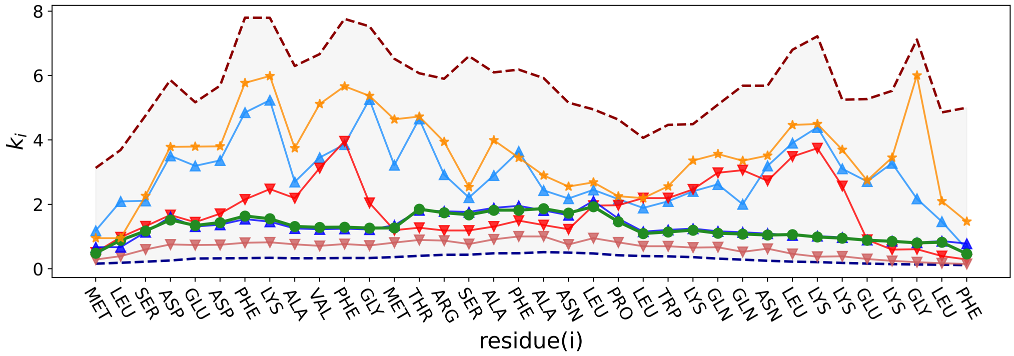


**Figure S6.** Distribution of the local force constants at times $t_0$ (bold green), $t_1$ (ligth blue), $t_2$ (red), $t_3$ (brown), $t_4$ (dark blue) and $t_5$ (orange) indicated in Figure S3 and discussed in the text. The grey area limited by dashed lines represents the range of values observed in the MD trajectory.

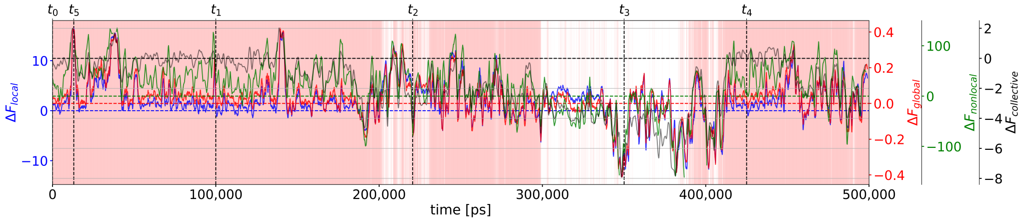


**(a)**

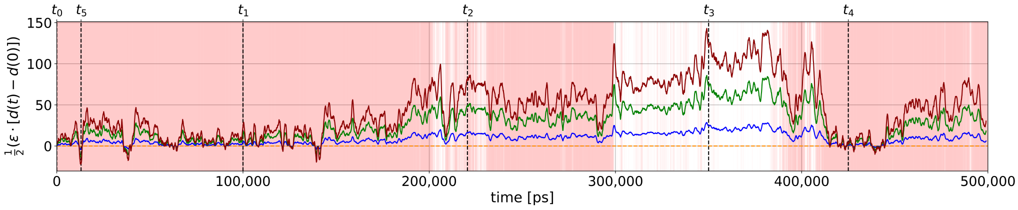


**(b)**

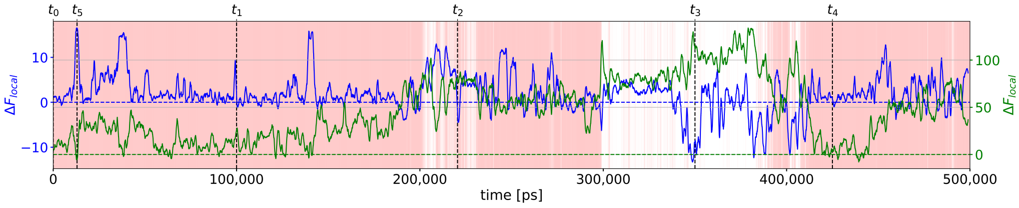


**(c)**

**Figure S7.** Free-energy graph calculations for the trajectory of Figure S1. (a) Local (blue), nonlocal (green), global (red), and collective (black) free-energy with $\epsilon = 0$. Horizontal dashed lines indicate the zero baselines of the free-energies with the corresponding colors. (b) Enthalpy term of the free-energy graph with $\epsilon = -1$ (blue), $\epsilon = -3$ (green), $\epsilon = -5$ (dark red). Horizontal dashed line indicates the zero baseline. $d(t) \equiv \sum_i d_i(t)$ and $d(0) \equiv \sum_i d_i(0)$. (c) Local free-energy with $\epsilon = 0$ (blue) and $\epsilon = -5$ (green). Folded regions are indicated by red vertical lines as in Figure S1. Horizontal dashed lines indicate the zero baselines of the free-energies with the corresponding colors.

## S4. Generalized Randić Theorem and Relation with Compliance

Here we demonstrate that the Randić resistance[2] between two nodes of an elastic network, i.e. the inverse of the nonlocal force constant, can be interpreted as the linear response of the nodes to a couple of opposite unit forces as stated in the main text. This implies that the Randić resistance is identical to the mechanical compliance of a pair of nodes.

First, the Randić theorem for electrical network can be generalized by considering the following linear response equation

$$\sum_{k=1}^{n} L_{ik} a_k = b_i \tag{S12}$$

where $L_{ik}$ is a symmetrical matrix obeying the following sum rule

$$\begin{aligned} \sum_{k=1}^{n} L_{ik} &= 0 \; \forall \, i \\ \sum_{i=1}^{n} L_{ik} &= 0 \; \forall \, k \end{aligned} \tag{S13}$$

and represents the linear response of a network where each node is a vertex of a graph representing or not an actual physical system. The $a_k$ and $b_i$ are components of vectors representing the responses of nodes and their excitations, respectively. Because of Equation (S13), the matrix $L$ is singular. The Moore-Penrose generalized inverse is

$$L^{-1}_{ik} = \sum_{l=2}^{n} \frac{1}{\lambda_l} e_l(i) e_l(k) \tag{S14}$$

where $\lambda_l$ are the eigenvalues of $L$ sorted by increasing values. The $e_l(i)$ are the components of the $lth$ eigenvector of $L$. Because of Equation (S13), we have $\lambda_1 = 0$ and $e_1(i) = C$ where $C$ is a constant. Because of the completness of the basis set of $L$, we deduce

$$\sum_{k=1}^{n} L^{-1}_{ik} L_{km} = \delta_{im} - C^2 \tag{S15}$$

Multiplying Equation (S12) by $L^{-1}$ and using Equation (S15) for two different nodes, we find

$$\begin{aligned} a_m - C^2 \sum_{k=1}^{n} a_k &= \sum_{i=1}^{n} L^{-1}_{mi} b_i \\ a_{m'} - C^2 \sum_{k=1}^{n} a_k &= \sum_{i=1}^{n} L^{-1}_{m'i} b_i \end{aligned} \tag{S16}$$

Substraction of the two lines of Equation (S16) leads to a generalized Randić theorem

$$a_m - a_{m'} = \sum_{i=1}^{n} \left[ L^{-1}_{mi} - L^{-1}_{m'i} \right] b_i \tag{S17}$$

Equation (S17) is a general equation where we examine the response of two nodes of a network to a set of excitations represented by the vector $\overrightarrow{b}$ of length $n$. For a particular excitation defined as follows:

$$b_i \equiv b(\delta_{mi} - \delta_{m'i}) \text{ for } i = 1, 2 ... n \tag{S18}$$

Equation (S17) is reduced to the Randić theorem[2]:

$$\begin{aligned} a_m - a'_m &= \Omega_{mm'} b \\ \Omega_{mm'} &= L^{-1}_{mm} + L^{-1}_{m'm'} - 2L^{-1}_{mm'} \end{aligned} \tag{S19}$$

where $\Omega_{mm'}$ is the original Randić resistance[2] if $\vec{a}$ is the current and $\vec{b}$ is the voltage. If $L$ represents the Laplacian of a connected, undirected and simple graph, the Randić resistance can be related to the nonlocal force constant of an atom pair of a linear elastic chain by $\Omega_{mm'} = 1/K_{mm'}$ as stated in the main text (see Equations (31) and (32) in the main text).

The resistance $\Omega_{mm'}$ also is exactly the compliance $C_{mm'}$[3] of an atom pair $(m, m')$ of an elastic linear chain. Indeed, from Equation (9) in the main text, we have:

$$a_m - a'_m = \Omega_{mm'} b$$
$$\Omega_{mm'} = \left(\phi^{-1}_{mm} + \phi^{-1}_{m'm'} - 2\phi^{-1}_{mm'}\right) = C_{mm'} \tag{S20}$$

where $\phi$ is the Hessian of the linear chain, $a_m$ and $a'_m$ are the atomic displacements of the atoms $m$ and $m'$ and $b$ is the amplitude of the couple of forces applied to this pair of sites.

It is worth noting that the relation with the compliance computed numerically for a three-dimensional elastic network representing the protein (see Ref. [3]) is in fact a tensorial problem. The Hessian and the response matrix $L$ are $3n$x$3n$ matrix and $\vec{a}$ and $\vec{b}$ are vectors of length $3n$. Therefore the compliance (Randić resistance or its inverse, the nonlocal force constant) is in fact a 3x3 tensor for a couple of forces:

$$a^{\alpha}_{m} - a^{\alpha}_{m'} = \sum_{\beta} \Omega^{\alpha\beta}_{mm'} b^{\beta}$$
$$\Omega^{\alpha\beta}_{mm'} = \left(\phi^{-1,\alpha\beta}_{mm} + \phi^{-1,\alpha\beta}_{m'm'} - 2\phi^{-1,\alpha\beta}_{mm'}\right) \tag{S21}$$

with $\alpha$, $\beta = x, y$ or $z$.

The exact relation between the Randić resistance and the *scalar* compliance $C_{mm'}$ of an atom of a *three-dimensional* elastic network[3] is as follows. The authors defined a scalar compliance by applying a couple of forces *along the direction of the vector joining two atoms in a protein*, i.e. in the direction $\vec{r}_m - \vec{r}_{m'}$ where $\vec{r}_m$ and $\vec{r}_{m'}$ are the positions of atoms $m$ and $m'$ in the structure when no forces are applied. In this case, we have $b^{\beta} = b\left(r^{\beta}_{m} - r^{\beta}_{m'}\right)$ with $b$ the amplitude of the couple of forces. if $\vec{a}_m$ and $\vec{a}_{m'}$ are the atomic displacements induced by this couple of forces, Equation (S21) reads

$$a^{\alpha}_{m} - a^{\alpha}_{m'} = \sum_{\beta} \Omega^{\alpha\beta}_{mm'} b\left(r^{\beta}_{m} - r^{\beta}_{m'}\right)$$

From Equation (12) of Ref. [3] defining $C_{mm'}$ in this case, we find the exact relation between this quantity and the Randić resistance tensor (or its inverse, the nonlocal force constant tensor)

$$C_{mm'} \equiv \sum_{\alpha} (a^{\alpha}_{m} - a^{\alpha}_{m'})(r^{\alpha}_{m} - r^{\alpha}_{m'}) \tag{S22}$$
$$C_{mm'} = \sum_{\alpha}\sum_{\beta} (r^{\alpha}_{m} - r^{\alpha}_{m'})\Omega^{\alpha\beta}_{mm'}\left(r^{\beta}_{m} - r^{\beta}_{m'}\right) \tag{S23}$$


1. Nicolaï, A.; Delarue, P.; Senet, P. Intrinsic Localized Modes in Proteins. *Scientific Reports* **2015**, *5*, 18128. Number: 1 Publisher: Nature Publishing Group, https://doi.org/10.1038/srep18128.
2. Klein, D.J.; Randić, M. Resistance distance. *Journal of Mathematical Chemistry* **1993**, *12*, 81–95. https://doi.org/10.1007/BF01164627.
3. Scaramozzino, D.; Khade, P.M.; Jernigan, R.L.; Lacidogna, G.; Carpinteri, A. Structural compliance: A new metric for protein flexibility. *Proteins: Structure, Function, and Bioinformatics*

**2020**, *88*, 1482–1492. _eprint: https://onlinelibrary.wiley.com/doi/pdf/10.1002/prot.25968, https://doi.org/10.1002/prot.25968.
